# Why nutrition education is inadequate in the medical curriculum: a qualitative study of students’ perspectives on barriers and strategies

**DOI:** 10.1186/s12909-018-1130-5

**Published:** 2018-02-12

**Authors:** Victor Mogre, Fred C. J. Stevens, Paul A. Aryee, Anthony Amalba, Albert J. J. A. Scherpbier

**Affiliations:** 1 Department of Health Professions Education and Innovative Learning, School of Medicine and Health Sciences, University for Development Studies, P. O. Box TL, 1883 Tamale, Ghana; 2 School of Health Professions Education, Faculty of Health, Medicine and Life Sciences, Maastricht University, Maastricht, The Netherlands; 3 Department of Nutritional Sciences, School of Allied Health Sciences, University for Development Studies, Tamale, Ghana

**Keywords:** Nutrition education, Medical students, Barriers, Strategies, Curriculum, Qualitative research

## Abstract

**Background:**

The provision of nutrition care by doctors is important in promoting healthy dietary habits, and such interventions can lead to reductions in disease morbidity, mortality, and medical costs. However, medical students and doctors report inadequate nutrition education and preparedness during their training at school. Previous studies investigating the inadequacy of nutrition education have not sufficiently evaluated the perspectives of students. In this study, students’ perspectives on doctors’ role in nutrition care, perceived barriers, and strategies to improve nutrition educational experiences are explored.

**Methods:**

A total of 23 undergraduate clinical level medical students at the 5th to final year in the School of Medicine and Health Sciences of the University for Development Studies in Ghana were purposefully selected to participate in semi-structured individual interviews. Students expressed their opinions and experiences regarding the inadequacy of nutrition education in the curriculum. Each interview was audio-recorded and later transcribed verbatim. Using the constant comparison method, key themes were identified from the data and analysis was done simultaneously with data collection.

**Results:**

Students opined that doctors have an important role to play in providing nutrition care to their patients. However, they felt their nutrition education was inadequate due to lack of priority for nutrition education, lack of faculty to provide nutrition education, poor application of nutrition science to clinical practice and poor collaboration with nutrition professionals. Students opined that their nutrition educational experiences will be improved if the following strategies were implemented: adoption of innovative teaching and learning strategies, early and comprehensive incorporation of nutrition as a theme throughout the curriculum, increasing awareness on the importance of nutrition education, reviewing and revision of the curriculum to incorporate nutrition, and involving nutrition/dietician specialists in medical education.

**Conclusion:**

Though students considered nutrition care as an important role for doctors they felt incapacitated by non-prioritisation of nutrition education, lack of faculty for teaching of nutrition education, poor application of nutrition science and poor collaboration with nutrition professionals. Incorporation of nutrition as a theme in medical education, improving collaboration, advocacy and creating enabling environments for nutrition education could address some of the barriers to nutrition education.

## Background

There is ample evidence that nutrition interventions can decrease morbidity, mortality, human suffering, and medical costs [1–4]. Given the cost-effectiveness of disease prevention through nutrition over pharmacological treatment, the availability of practicing doctors with adequate knowledge, attitudes and skills in nutrition is very essential [5]. Studies have reported that if doctors gave nutrition advice to their patients the incidence of nutrition-related diseases will decline [6, 7]. However, most doctors usually miss the opportunity to provide nutrition care to patients in the general practice setting and often refer patients to hospital dieticians or nutritionists, that is if these are available [8, 9]. Research suggests that practicing doctors lack sufficient nutrition care competencies to provide dietary advice to their patients [10–15].

Indeed, there is an unfilled role for medical education in preparing doctors to provide nutrition care to patients [16]. Several studies investigating the situation of nutrition education in the medical curriculum have found both practicing doctors and medical students reporting inadequate nutrition education during medical school [14, 17–21].

Several barriers to inadequate nutrition education have been reported largely in the form of symposiums, commentaries, essays and special articles [8, 22–25]. Few studies have systematically investigated the issue [5, 26–28]. These studies generally echo the perspectives of teachers or practicing doctors. A number of strategies to improving nutrition education in the medical curriculum have also been discussed by some of these studies [8, 22, 23]. However, the perspectives of students are unclear.

This is an important issue to investigate, as students are likely to bring unique and valuable perspectives on the inadequacy of nutrition education in their curriculum. Their views should be considered in efforts to improve nutrition education in the medical curriculum.

The study therefore aimed to explore students’ perceptions of doctors’ role in nutrition care, and barriers that prevent effective nutrition education and strategies that could potentially increase the effectiveness of nutrition education in the medical curriculum. The following research questions were investigated

1. What are the roles of doctors in the provision of nutrition care in the general practice setting?
2. Why is nutrition education inadequate in the medical curriculum?
3. How could nutrition education be improved in the medical curriculum?

## Methods

### Theoretical orientation

Our research was informed by two closely related theories [29]: social learning theory and social cognitive theory. Arguably, delivery of nutrition care by health care providers is a behaviour demonstrated in the social context of the workplace settings which is influenced by observing and modelling the behaviours, attitudes, and emotional reactions of others (e.g. superiors) [30]. Also embedded in our study is the social cognitive theory of learning which emphasizes how cognitive, behavioural, personal, and environmental factors interact to determine motivation and behaviour [31]. Nutrition education in the medical school setting is a learning process that is influenced by factors within the academic environment relating to the quantity, quality and nutrition content in the curriculum as well as the teaching and learning methods employed and the reinforcement experienced by the individual and by others.

### Methodology

Our research is within the constructivists’ research paradigm. In constructivism, knowledge is socially constructed and realities are generated by the interaction of social, cultural and interpersonal factors [32, 33]. Accordingly, there are multiple accounts of realities and meaning is realised through mutual interactions between the researcher and participants and the context of the research [34]. In this research approach, identification of the background and assumptions of the researcher and how they may influence the data collection and analysis is important and should be reflected upon by the researcher and shared with readers to facilitate interpretation of the research findings [35, 36]. VM has a nutrition background and teaches in the medical school. His co-authors have significant experience in medical education (AJJA, FCJS), qualitative research (AJJA, FCJS), nutrition (PAA), pharmacology (AA) and also teach in the medical school (AJJA, FCJS, AA, PAA).

### Setting, participants and procedures

We adopted a purposive sampling to select the study participants. All participants were selected from the University for Development Studies, School of Medicine and Health Sciences (UDS-SMHS), which runs a problem-based undergraduate medical curriculum. In the first 3 years, students are taken through the basic sciences using system-based blocks. The next year focuses on the learning of pathology and patho-physiology of diseases. Although students have some early patient encounters, the first 4 years are usually non-clinical (preclinical). In preparing students to qualify as doctors, the remaining 3 years focus on the clinical application of principles learned in the preclinical years and the creation of opportunities for students to learn from real patients at the hospital through departmental clinical rotations. The UDS-SMHS also runs a community-based education and service (COBES) programme in which students live, learn and provide service in rural communities of Ghana for a period of 4 weeks per year during year 2 to 4. It is during the preclinical years that majority of nutrition education is learned.

Our participants were therefore clinical level students, who had covered over 50% of the curriculum. Our choice of participants and sampling procedure was informed by our aim to select participants who will provide rich information [36, 37] regarding nutrition education in the medical curriculum.

VM contacted all prospective participants through face-to-face meetings after usual lectures. During such meetings, the purpose of the study was explained and participants were also informed that the interview will be audio-recorded. They were assured of the confidentiality of the recordings. Participation was voluntary and both written and verbal informed consent was obtained from all participants who agreed to participate in the study. The institutional review board of the Navrongo Health Research Institute approved the research protocol.

### Semi-structured interviews

All data was collected using semi-structured individual interviews. Semi-structured interviews are frequently used in healthcare and provide the researcher the opportunity to have guidance on areas to explore while allowing for some flexibility to enable discovery or elaboration [38, 39]. All interviews were conducted by VM to ensure uniformity. Interviews were informed by open ended questions to explore values, attitudes, experiences, opinions, and behaviours of participants regarding nutrition education in the medical curriculum (See Appendix for interview guide). Interviews provided time and scope for participants to give detail and in depth about their opinions regarding nutrition education in the medical curriculum, allowing for their understanding and point of view rather than assuming generalizations. The questions were derived from the literature [27, 40]. During the interviews VM probed and sought for clarification or elaboration of participants’ responses as needed. Opportunities were provided for participants to voice out unsolicited opinions and experiences.

The questions were evaluated by all members of the team who have varying levels of experience in qualitative research, medical education and nutrition. We pilot tested the semi-structured interviews on a group of 8 students to ensure clarity and understanding of the questions. During this process participants provided feedback regarding the structure of the questions and also identified areas that needed clarification. AA observed these pilot interview sessions, providing the opportunity to train and give feedback. The data generated from the pilot sessions were not included into the main data analysis.

### Data analysis

All interviews were transcribed verbatim without identifying information by a trained research assistant, and entered into MAXQDA (version 12), a qualitative data analysis software. All subsequent data analyses were conducted using a comparative strategy [41, 42]. Coding was performed by VM, who initially applied as many codes as needed for each transcript and inductively renamed, reorganised and redefined codes as required within categories. Another member of the team, AA went through the same process to evaluate VM’s categorisation. VM and AA then compared and discussed their findings until a consensus was achieved. Disagreements were resolved through discussions and adjudication by AA or FCJS if needed. To raise the analytic level from categorical to conceptual, axial coding was performed to identify dominating themes and to explore relationship among these themes. All members of the research team discussed the results of the analysis process until a consensus was established. Data collection and analysis were conducted simultaneously until thematic saturation was realised.

## Results

Twenty-three students participated in semi-structured interviews lasting 10 to 20 min each during the study period, July to September, 2015. This number was arrived at after the point of saturation and further data collection and/or analysis was unnecessary. Table 1 presents the demographic characteristics of the participants.

**Table 1:** Demographic characteristics of participants (*n* = 23)

Variable	Frequency	**%**
Mean Age (SD)	25.0 (2.76)	
Age category		
Below 25	14	61
25 +	9	39
Gender		
Male	14	61
Female	9	39
Clinical year		
Year one	7	30
Year two	9	39
Year three	7	30

Four main themes emerged from the data: role of doctors in nutrition care, adequacy of nutrition education, barriers to nutrition education, and strategies to increase and improve nutrition education. Illustrative quotes are provided to substantiate each of these themes. Statements represent the views of students and not the authors.

### Theme 1: The role of doctors and nutrition care

The majority of the students felt that doctors had an important role to play in providing nutrition care to patients although they recognised that nutritionists and dieticians were trained to perform such functions. They considered the role of the doctor to be one that is supportive or complementary to that of the nutritionist/dietician. The main roles identified by the students are shown in list 1.

**List 1: Roles doctors could play in nutrition care**

- Nutrition advice/education
- Supporting patients to follow healthful diets
- Collaborating with nutritionists/dieticians
- Monitoring progress of nutrition care
- Nutrition diagnosis
- Referring patients to nutritionists
- Advocating for nutrition care

*“They [doctors] should also play a part in the health education of the patient. So when they see a patient of any kind they have to advise them on their nutrition, diet and other behaviours that affect their nutrition and all of that.”*

Students believed that the doctor should be the first to provide basic nutrition care and refer the patient to a nutritionist/dietician for specialised care if need be.

*“When they [patients] come to the hospital, the doctor has the first encounter with them. The doctor could begin to provide nutrition care based on his/her minimal knowledge in nutrition. Then afterwards if he/she sees that there is still more to be done, then he/she can refer the patient to the nutritionist or the dietician who are specially trained to provide nutrition care.”*

In order to assess whether medical students considered themselves to be responsible for the nutrition care of their patients when they become doctors, they were asked a hypothetical question about how they will react if a diabetic patient sought dietary advice from them in the general practice setting. All of the students said they will feel obligated and enthusiastic to provide such dietary advice to the patient.

*“Oh I will feel happy to assist the patient. As such I will tell the patient the causes of diabetes and what to do to avoid [prevent] it. I will advise the patient to avoid excessive intake of sugar.”*

### Theme 2: Adequacy of nutrition education

Although students felt that nutrition care was important and felt obligated to provide it to patients, majority of them felt unconfident in their ability to provide such care to their patients.

*“Well I think it is the right thing to find out how they [patients] manage their nutrition based on their condition. But looking at the kind of training that we [students] also have, you realize that much attention is not given to nutrition. So if a patient should ask me at this moment about nutritional status or let’s say about their nutritional situation, I might not feel adequate enough to answer.”*

In addition, almost all the students considered the quality and quantity of their nutrition education to be inadequate.

*“For me I think… even though we have done something very little concerning nutrition, it is as if we [students] have not done anything. We [students] have been given very little training when it comes to nutrition.”*

It is thus unsurprising that students felt they required further education in nutrition although they were in the senior years of their medical education.

*“I think there is more to be done because we [students] have not really had more lectures on it. We [students] have not been taken through a lot on nutrition; it is just a little bit of everything like small, small, small. So I think it would be better if we [students] are given more education on nutrition before we are able to come out.”*

In order to gain more knowledge and skills in nutrition some students undertook self-directed learning and also consulted nutrition departments at the hospital on their own volition to acquire knowledge and skills in nutrition care.

*“Because we [students] realized that our nutrition education was inadequate, when we [students] came here we had to go to the nutrition department to kind of like talk to them to take us through how they managed their [nutrition department] malnutrition cases”.*

### Theme 3: Barriers to adequate nutrition education

Students identified a number of barriers contributing to the inadequacy in their nutrition education. These barriers were classified into three subthemes: personal, interpersonal and environmental barriers as shown in Table 2. In accordance with the descriptions of Williams et al. [43], personal barriers refers to factors related to the individual, whiles interpersonal barriers are attributable to relationship between two or more individuals. Contextual factors that may influence nutrition education are described as environmental barriers. Next we describe further some of these barriers.

**Table 2:** Barriers to nutrition education generated from the data

Category	Barrier
Personal	
	Poor translation of nutrition science to clinical practice
	Perception that nutrition care is not the responsibility of doctors
	Faculty’s lack of knowledge in clinical nutrition
	Faculty’s lack of motivation to teach nutrition
Interpersonal	
	Poor collaboration with nutrition professionals in medical education
Environmental	
	Lack of priority for nutrition
	Lack of faculty to provide nutrition education
	Poor integration of nutrition as a theme throughout the curriculum
	Already overcrowded curriculum
	Time constraints
	Lack of role models to model nutrition care

#### Poor translation of nutrition science to clinical practice

Most students felt that the application of nutrition science to clinical practice was poor in most of the already limited nutrition lectures they received, especially during their preclinical training. The apparent lack of application of nutrition science to clinical practice made it difficult for them to appreciate the relationship between nutrition and health and to apply nutrition during their clinical training.

*“Though at the preclinical level we [students] were given a little bit knowledge about nutrition it was not practical. It was like kind of raw information. You come here [clinical training] then you realize that there is no correlation. Not correlation as such but here [clinical training] you realize that it demands you to apply.”*

Another student adds that *“the practical aspect of nutrition education has not really been taught or we are not exposed to it”.*

#### Perception that nutrition care is not the responsibility of doctors

Another important barrier identified by the students was the perception of faculty and curricula planners that nutrition care may not be the responsibility of doctors and as such did not see the need to include adequate content of nutrition education into the curriculum. According to the students, faculty and curricula planners considered nutrition care to be the role of nutritionists and thus the medical curriculum should concentrate on how to treat diseases.

*“May be the authorities’ feel it’s not an essential part of medical training. I don’t know but that is the way I see it”.*

*“May be the focus of the whole medical training is more on treating diseases.”*

*“I think because there are nutritionist who are specially trained to provide nutrition care”.*

#### Poor collaboration with nutrition professionals in medical education

Students were concerned that their nutrition education did not involve nutrition professionals thus presenting limited opportunities to collaborate to learn about nutrition. The poor collaboration made it difficult for them to appreciate the role of nutrition care in improving the clinical outcomes of patients and also do not foster inter-professional training to promote multidisciplinary care required in patient care.

*“If there can be more integration. For instance when we [students] have cases that are nutrition related, it is still being managed by medical professionals and doctors. I mean once in a while dieticians come but it is like off record or something. So if those who are directly involved in the nutrition sector can be integrated more. May be it will help. But right now they are separated, nutrition is on one side and medicine is on the other. So maybe if there is a problem that will be the area it is.”*

Students identified that nutrition professional were not involved in their training because they were not available i.e. they were in short supply.

#### Lack of priority for nutrition education

Majority of the students felt that the medical curriculum concentrates largely on anatomy, physiology and pathology with a little attention for nutrition. The students felt strongly that nutrition has not been given the needed attention and priority that it deserved.

*“Like we have anatomy, physiology and the other basic sciences incorporated, we don’t actually have a course on nutrition incorporated into the courses that we do. I think that is the major issue”.*

*“It’s also because nutrition is not a priority in the medical program and hence have been given less attention.”*

According to the students, the lack of priority for nutrition education has resulted in the limited contact hours allocated for nutrition, inadequate content for nutrition in the medical curriculum and poor integration of nutrition education as a theme throughout the entire medical curriculum.

#### Lack of faculty to teach nutrition

Students were also concerned about the lack of trained faculty to provide nutrition education.

*“May be it could also be that we don’t have the people around to actually, I mean teach us or take us through nutrition.”*

*“I think sometimes the lecturers may not be available to give a lecture on that [nutrition].”*

A number of students also felt that faculty does not have the needed knowledge and skills to provide students with the required nutrition education.

*“May be I think it is….Because those taking you, they themselves they don’t have the clinical knowledge. It is like they just read raw nutrition [theoretical aspect of nutrition] and that’s how they also passed it on to you.”*

### Theme 4: Strategies to improve nutrition education

Having recognized the inadequacy of their nutrition education and the contributing barriers, students provided several important and insightful recommendations that could improve nutrition education.

#### Increase lectures, tutorials and self-study time for nutrition education

To increase the contact hours and nutrition content in the curriculum students suggested that more lecture topics on nutrition should be included into the curriculum.

*“Not really as in a full course but may be some few lectures in nutrition. I don’t think we need so much but some few things that you need on the job that to help you, that is very important. I think that aspect could be incorporated into the training, it will help.”*

Others also suggested that nutrition training could be improved by incorporating nutrition topics into their tutorials to promote self-directed learning.

*“I think that we can also introduce them into our tutorial topics because sometimes for most of the students tutorials really help. You read a lot before you come so you can introduce some of them into our tutorial topics”.*

Concerning the promotion of self-directed learning and to cater for the overcrowded curriculum, students suggested that articles on nutrition could be given to them to read to promote their understanding of nutrition concepts. The self-study time for students could also be increased to grant students the opportunity to read further on nutrition. In addition, students suggested the organization of symposiums on nutrition in which research papers in nutrition could be presented in order to increase their interest and understanding of nutrition.

*“Well it’s very dicey because there is time issue and stuff. But if we could get articles or make symposiums where we can teach nutrition courses and make it lively because nutrition is kind of like difficult. So if we make it motivational they will be able to come out and get the knowledge about nutrition so that they can educate the people.”*

#### Early incorporation of nutrition as a theme and throughout the entire curriculum

As evidenced by the following code, students felt that their knowledge and skills in nutrition could be improved if nutrition as a theme was incorporated early in their training and maintained throughout the entire curriculum.

*“Looking at the early [preclinical] aspect of the training, they can incorporate nutrition subjects there, nutritional modules or topics and things like that especially at the very early aspects of it. So that people [students] will take interest in that and probably might grow their knowledge as they go along the PBL training.”*

#### Increasing awareness on the importance of nutrition education in the medical curriculum

Some students opined that nutrition education will be improved if those in charge were educated on the importance and need for nutrition education. Students believed that if faculty and curriculum planners could be convinced of the fact that nutrition is an important role of the doctor, this might change their perception regarding nutrition and increase attention and priority for nutrition. An increase in attention and priority for nutrition education may result in an increase in the content and contact hours for nutrition.

*“I think if they can realize that it is also very very important then they can increase the amount of time that is spent on that [nutrition education]”.*

#### Reviewing and revision of the curriculum to incorporate nutrition

Students believed that for nutrition education to be improved there is the need for faculty and curriculum planners to review the entire curriculum to identify students nutrition educational needs and how and where those needs could be met and incorporated into the curriculum. Students recognised that such revisions could not be effected during their time in the medical school but could be beneficial to those in the succeeding years in medical school.

*“That is what I previously stated that they should revise our curriculum and find a place to put nutrition so that at least we [students] will have knowledge about it. At least at this level I should have had enough knowledge about nutrition. So if they put it there those following us can benefit. Right now if they can add it to our curriculum, I think it will be enough.”*

#### Involving nutrition/dietician specialists

Students opined that nutritionists or dieticians should be actively involved in their training. This should take the following forms:

- Clinical sessions in which nutritionists/dieticians will present nutrition cases
- Opportunities to consult nutritionist/dieticians for relevant nutrition topics/content
- Including the nutrition department into clinical rotations
- Seminars and lectures presented by nutritionists/dieticians

*“It [nutrition education] should be added to the medical school’s curriculum and we [students] should actually...ehmm if they have a nutrition department, we should actually be exposed to nutrition” “Even in the hospital we [students] can have nutritionist being involved in our training on the ward especially when we [students] are managing patients so that we can have more time with the nutritionist”.*

## Discussion

### General discussion and doctors’ roles in nutrition care

Undergraduate clinical level medical students believed medical doctors have a responsibility to provide nutrition care to their patients in the general practice setting. They believe doctors’ role regarding nutrition care should be one of identifying patients in need of nutrition care, providing first line nutrition management, advocating nutrition care for patients, referring patients to nutritionists/dieticians for specialist care and reinforcing nutrition care provided by nutritionists/dieticians. These students felt their current nutrition education was inadequate due to barriers such as lack of priority for nutrition education, poor application of nutrition to clinical practice, an already overcrowded medical curriculum, lack of faculty trained in nutrition and poor collaboration with nutrition professionals in the teaching and learning of nutrition. Students recognised the need to increase awareness on the relevance and need for nutrition education in the medical curriculum among faculty and curriculum planners and to undertake a revision of the medical curriculum to identify nutrition educational needs and avenues to meet such needs as well as recognising the need to involve nutritionists/dieticians in their training.

Student’s views that medical doctors have an important role to play regarding nutrition care are similar to those expressed by medical educators, practicing doctors [27, 44] and students [45] of previous studies from other parts of the world.

### Barriers to nutrition education and suggested strategies

Consistent with previous findings, students were concerned that their nutrition education was inadequate [17, 45–50].

Although an already overcrowded curriculum and inadequate contact hours/content for nutrition education were frequently described as barriers to effective nutrition education, some of the students believed that this was more an issue of low priority and attention for nutrition education. According to these students if nutrition was considered an important responsibility of the medical doctor, faculty and curriculum planners will find avenues within the curriculum to include nutrition. There is thus the need to increase faculty and curriculum planners’ awareness on the relevance and need for nutrition education in the medical curriculum.

Students’ suggestion of integrating nutrition as a theme throughout the entire curriculum to cater for barriers such as inadequate contact hours/content for nutrition and overcrowded curriculum is consistent with best practices for improving nutrition education [5, 51]. Integration of nutrition as a theme in the medical curriculum has been shown to be effective in reducing students’ perception of the inadequacy of the amount of nutrition taught to them and improving nutrition clinical skills as assessed by Objectively Structured Clinical Examination (OSCE) scores [52]. The integration of nutrition as a theme throughout the curriculum should be comprehensive including the early stages of preclinical training up to clinical training and continued to postgraduate medical education [5, 8, 24, 53].

Fundamental to the feasibility of integrating nutrition as a theme throughout the medical curriculum is the need to undertake a review of the entire curriculum. This provides opportunities to look into the formal and informal/hidden curriculum to identify gaps and avenues to integrate nutrition education and improve the learning environment [5, 54].

Given that multidisciplinary teaching is a key focus of education guidelines for future health professionals [55] it is consistent that students’ recognized poor collaboration with nutrition and other health professionals in their nutrition education as an important barrier. The apparent poor collaboration may not give students the opportunity to realise the multidisciplinary nature of nutrition care [56] and will hinder inter-professional development and collaboration required for clinical care [57]. The identification and integration of nutrition content into the curriculum may also be hampered if nutrition professionals are not involved in the planning and development of the medical curricula. Collaborating with nutrition and other relevant health professionals has been found to be effective in improving the status of nutrition education in the medical curriculum [56]. Reflecting on the success of a nutrition education initiative at the University of Cambridge, Ball et al. [56] asserts that the multidisciplinary nature of the programme contributed to its success. The multidisciplinary team included medical practitioners, dieticians, nutritionists, and nurses in the delivery and evaluation of nutrition education sessions. This multidisciplinary nature was recognized at two levels: during the development of the intervention and implementation of the teaching and learning strategies of the intervention. This approach has also been widely encouraged by other institutions to model the contribution of health professionals in addressing nutrition in patient practice [58, 59].

The students suggestion of providing them with nutrition articles and the organisation of nutrition symposiums and seminars to address the barriers of overcrowded and inadequate curriculum are widely accepted innovative teaching and learning strategies of improving nutrition education [60, 61]. These strategies are more likely to be adopted by students and could result in improved nutrition education given that they perceive nutrition to be important to their future practice as medical doctors.

The lack of faculty for nutrition education is an important barrier that has been identified in previous findings [5, 8, 24]. Closely linked to this barrier is faculty’s lack of interest in nutrition education. These barriers are fundamental to the effectiveness of nutrition education in the sense that if faculty trained in nutrition are lacking, increases in contact hours and nutrition content and integration of nutrition as a theme in the curriculum may not yield the needed results. The availability of faculty trained in nutrition may facilitate identification of appropriate nutrition content, coordination of teaching and learning activities in nutrition, promotion of active participation in nutrition education activities by both faculty and students, translation of nutrition science to clinical practice, and promotion of role modelling of nutrition care at the hospital [52, 61–64].

### Strengths and limitations

This is the first study to qualitatively explore students’ perceptions of nutrition education, barriers and strategies as far as we know. It adds to the existing literature that nutrition education is inadequate and several barriers are contributing to the current situation. Also, the findings identify a number of strategies that could be relied upon to improve nutrition education. An important novelty of the methods of this study is the reliance on students’ perspectives that hitherto may not be considered as obvious sources of information regarding this topic. The students provided important barriers and strategies that are comparable to those reported by medical educators and practicing doctors. It is yet additional evidence supporting the widely accepted recognition of students as important stakeholders of the curriculum who could be relied upon to improve the learning environment.

This study is not without limitations. Our adoption of non-probability and convenience sampling may result in the recruitment of students with an interest in nutrition, generating a potential source of sampling bias. However, this potential bias can be described as a strength rather than a limitation in qualitative research that propagates for the collection of data from individuals considered as rich sources of information [27]. We recruited students from only one medical school, thus limiting the generalizability of our findings. The opinions expressed by these students, only grant one aspect of the situation of nutrition education in the medical curriculum and may be insufficient in providing a holistic and complete understanding of the situation. Nevertheless, following a constructivists approach, that aims to develop a comprehensive understanding of the situation of nutrition education, their opinions are valuable insights. As a measure to reduce interviewer’ bias that may influence students’ opinions during the semi-structured interviews, we limited the interviews to questions and clarifications, where necessary and avoided expression of interviewer opinion. Our study explored students’ opinions regarding nutrition education but did not evaluate whether improving nutrition education will result in improved nutrition practice behaviour or enhance clinical outcomes.

### Implications and future research

The findings of this study provide data that could inform the planning and design of educational interventions to improve nutrition education. Future studies should investigate whether the removal of the identified barriers could result in improved nutrition education. It will be important for studies to evaluate the effectiveness of the strategies suggested by the students to improve nutrition education in the medical curriculum. In addition, future studies should investigate the influence of improved nutrition education on nutrition practice behaviour in the clinical setting.

## Conclusion

Students perceived nutrition care to be an important role and responsibility of medical doctors. They considered their current nutrition education to be inadequate due to personal, interpersonal and environmental barriers. A number of important strategies have been suggested by these students including incorporation of nutrition as a theme in the medical curriculum, collaboration, and advocacy and creating enabling environments for nutrition education.

## Appendix

### Interview guide

**Medical students**

Age

Gender

What is your level in medical school?

In your opinion, what is the role of doctors in providing nutrition care in the general practice setting?

In your opinion, which professionals have a role in giving nutrition care to patients? Probe further for reasons.

How would you feel if a patient suffering from diabetes asked you for nutrition advice in the general practice setting?

Could you tell me your experience of patients requesting for nutrition advice in the general practice?

In what ways might patient care be affected if all doctors were able to give nutrition advice for routine care?

How will you describe the status of nutrition education in your medical curriculum? Probe further

In what ways have you learned about nutrition, if at all? Probe further

What is responsible for the current status of nutrition in your medical curriculum? Probe further

In what ways do you think your medical program needs improvement in nutrition? Probe further

In what ways could nutrition be improved in your medical program? Probe further

## Abbreviations

(PBL/COBES): and UDS-SMHS University for Development Studies, School of Medicine and Health Sciences
NHRCIRB: Navrongo Health Research Centre Institutional Review Board
PBL/COBES: Problem-based learning/Community-based Education and Service
